# Effects of glucose metabolism during *in vitro* maturation on cytoplasmic maturation of mouse oocytes

**DOI:** 10.1038/srep20764

**Published:** 2016-02-09

**Authors:** Hong-Li Xie, Yan-Bo Wang, Guang-Zhong Jiao, De-Ling Kong, Qing Li, Hong Li, Liang-Liang Zheng, Jing-He Tan

**Affiliations:** 1 College of Animal Science and Veterinary Medicine, Shandong Agricultural University, Tai-an City 271018, P. R. China

## Abstract

Although there are many reports on the effect of glucose metabolism on oocyte nuclear maturation, there are few studies on its effect on ooplasmic maturation. By manipulating glucose metabolism pathways using a maturation medium that could support oocyte nuclear maturation but only a limited blastocyst formation without glucose, this study determined effects of glucose metabolism pathways on ooplasmic maturation. During maturation of cumulus-oocyte-complexes (COCs) with glucose, the presence of PPP inhibitor, DHEA or glycolysis inhibitor, iodoacetate significantly decreased blastocyst rates, intraoocyte glutathione and ATP. While blastocyst rates, GSH/GSSG ratio and NADPH were higher, ROS was lower significantly in COCs matured with iodoacetate than with DHEA. Fructose-6-phosphate overcame the inhibitory effect of DHEA on PPP. During maturation of COCs with pyruvate, electron transport inhibitor, rotenone or monocarboxylate transfer inhibitor, 4-CIN significantly decreased blastocyst rates. Cumulus-denuded oocytes had a limited capacity to use glucose or lactate, but they could use pyruvate to support maturation. In conclusion, whereas glycolysis promoted ooplasmic maturation mainly by supplying energy, PPP facilitated ooplasmic maturation to a greater extent by both reducing oxidative stress and supplying energy through providing fructose-6-phosphate for glycolysis. Pyruvate was transferred by monocarboxylate transporters and utilized through mitochondrial electron transport to sustain ooplasmic maturation.

Oocyte maturation includes nuclear maturation and cytoplasmic maturation^1^. During nuclear maturation, oocytes resume meiosis from prophase-I to germinal vesicle (GV) breakdown, undergo metaphase-I, and progress through anaphase-I / telophase-I to metaphase-II (MII) stage^2^. Cytoplasmic maturation includes all of the other changes within the oocyte, such as accumulation of mRNA and protein, reorganization of the cytoskeleton and organelles, and changes in cellular metabolism^3^. In other words, while nuclear maturation is manifested as resumption of the first meiosis and extrusion of the first polar body (PB1), cytoplasmic maturation refers to acquisition of the ability to complete pre-implantation development^4^,^5^,^6^. It is recognized that the developmental capacity of *in vitro* matured (IVM) oocytes is inferior to that of the *in vivo* matured (IVO) oocytes, due mainly to insufficient cytoplasmic maturation^7^,^8^,^9^,^10^,^11^,^12^,^13^.

Energy metabolism is crucial for oocyte maturation because progression through all the dynamic processes involved requires a lot of energy from various substrates including carbohydrates, amino acids and lipids^14^,^15^. Studies have suggested beneficial effects of glucose metabolism on oocyte maturation^16^,^17^,^18^,^19^,^20^,^21^,^22^. For example, resumption of meiosis is associated with elevated activities of glycolysis and PPP within the oocyte cytoplasm^18^,^23^,^24^,^25^. Increased metabolism of glucose through one or more metabolic pathways also occurs simultaneously with the progression of meiosis to MII of oocytes^16^,^20^,^26^. Furthermore, gonadotropin-induced meiosis is dependent upon the presence of glucose^27^,^28^.

Although some studies suggest that the positive effect of glucose is mediated by glycolytic production of pyruvate^24^,^27^,^28^, which can then be oxidized to generate the energy necessary for nuclear maturation, other data indicate that the glucose requirement for meiotic induction does not depend on its glycolysis to pyruvate^29^,^30^,^31^,^32^,^33^,^34^. Based on their finding that purine nucleotide-generating pathways participated in gonadotropin stimulation of meiotic maturation^35^, Downs *et al.*^36^ proposed that the pentose phosphate pathway (PPP) was the alternative pathway to glycolysis that mediated the positive action of glucose. Those authors tested this hypothesis and concluded that the glucose metabolism through PPP was involved in the meiotic induction through the generation of phosphoribosyl pyrophosphate (PRPP)^36^. However, they were unable to observe a rise in PRPP following the activation of PPP with phenazine ethosulfate or pyrroline-5-carboxylate. Furthermore, they arrested meiotic maturation with hypoxanthine, which was found to lower the level of PRPP in the same study. Thus, the role of PPP in the glucose regulation of oocyte function must be studied using a system not including hypoxanthine.

Although there have been numerous reports on the effect of glucose metabolism on oocyte nuclear maturation, studies on the effect of glucose metabolism on oocyte cytoplasmic maturation are limited. Whereas some studies observed that glucose metabolic activity during oocyte maturation was correlated well with embryo development after insemination *in vitro*^16^,^17^,^19^,^20^,^21^, the effect of glucose metabolism on oocyte cytoplasmic maturation has rarely been studied by manipulating glucose metabolism during maturation *in vitro*. Furthermore, in the few studies manipulating oocyte glucose metabolism *in vitro*^37^,^38^, the effect of glucose metabolism on cytoplasmic maturation was analyzed together with its effect on nuclear maturation. Thus, the pathways through which glucose metabolism supports oocyte nuclear maturation remain to be specified and the effect of glucose metabolism on ooplasmic maturation has yet to be determined in exclusive studies not involving nuclear maturation.

In the current study, a maturation medium that could support oocyte nuclear maturation in the absence of glucose but sustained a limited blastocyst formation without glucose was first formulated. By manipulating glucose metabolism pathways using such a maturation medium, we determined the effects of various glucose metabolism pathways on ooplasmic maturation exclusively without involving nuclear maturation.

## Results

### The formulation of maturation medium

To formulate a maturation medium that could support oocyte nuclear maturation in the absence of glucose but could not sustain blastocyst formation without glucose, COCs were matured with different concentrations of glucose, pyruvate, lactate or both lactate and glucose. The matured oocytes were then aged and Sr^2+^ activated for embryo development. When COCs were matured with 0.5 mM lactate without glucose, acceptable rates of nuclear maturation (about 80%) were obtained, but rates of blastocysts decreased significantly compared to those in oocytes matured with 5.6 mM glucose (Table 1). When COCs were matured with both lactate and glucose, however, blastocyst rates increased to the same level as in oocytes matured with 5.6 mM glucose. The results suggested that the simplified α-MEM medium containing 0.5 mM lactate was a maturation medium that could support acceptable nuclear maturation of COCs but only a limited blastocyst formation without glucose. Furthermore, when COCs were matured with optimal concentrations of lactate (5 mM) or pyruvate (2 mM), whereas blastocyst rates obtained with pyruvate were as high as those obtained with 5.6 mM glucose, those obtained with lactate were significantly lower (Table 1). This suggested that while pyruvate in the culture medium and from glycolysis was fully utilized by the oocyte, the lactate-derived pyruvate was not.

### Effects of glucose metabolism in cumulus cells on oocyte maturation

To determine the effect of glucose metabolism in cumulus cells on oocyte maturation, DOs were matured and activated. None of the DOs matured after culture with lactate or glucose alone or lactate + glucose, whereas 100% of the DOs matured after culture with pyruvate (Table 1). About 9% of the DOs matured with pyruvate developed into blastocysts. Whereas only 6% of the DOs cultured with glucose alone underwent GVBD, about 25% of the DOs cultured with lactate or lactate + glucose completed GVBD. The results suggested that DOs had a limited ability to metabolize glucose, but they could use pyruvate to support cytoplasmic maturation and use lactate to support some nuclear maturation.

### Effects of inhibiting PPP or glycolysis during maturation on the developmental potential of mouse oocytes

COCs were matured with 5.6 mM glucose, 0.5 mM lactate and different concentrations of DHEA or iodoacetate, and the matured oocytes were aged and Sr^2+^ activated for embryo development. Although rates of activation and 4-cell embryos did not differ, rates of blastocysts decreased significantly in oocytes matured in the presence of either 150 μM DHEA or 1.5 μM iodoacetate compared to those in control oocytes matured without inhibitors (Table 2). The results suggested that both PPP and glycolysis were essential for cytoplasmic maturation of mouse oocytes.

### The DHEA- or iodoacetate-induced impairment of oocyte cytoplasmic maturation was not due to drug toxicity

To rule out the possibility that the inhibitors might impair oocyte developmental potential by their toxicity, COCs were matured with 2 mM pyruvate alone or with different concentrations of DHEA or iodoacetate. The matured oocytes were then aged and Sr^2+^ activated for embryo development. Blastocyst rates did not differ whether oocytes were matured with or without 150-μM DHEA or 1.5-μM iodoacetate, but decreased significantly when iodoacetate increased to 2 or 3 μM (Table 3). The results suggested that 150-μM DHEA or 1.5-μM iodoacetate that had successfully inhibited glucose metabolism in the above experiments was nontoxic to mouse oocytes.

### Effects of inhibiting PPP or glycolysis during oocyte maturation on GSH reserve and redox potential

**Figure 1:**
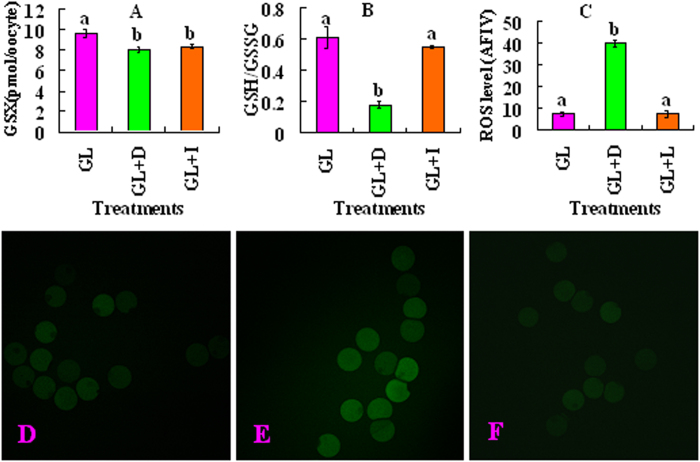
Concentration of GSX, the ratio of GSH/GSSG and levels of ROS in mouse oocytes after COCs maturation in the simplified α-MEM containing 5.6-mM glucose and 0.5-mM lactate (GL) in the absence or presence (+) of 150-μM DHEA (D) or 1.5-μM iodoacetate (I). Panel A and B show concentration of GSX and the ratio of GSH/GSSG, respectively. Each treatment was repeated 3 times with each replicate containing about 25 oocytes. Panel C shows levels of ROS. The concentrations of ROS were expressed as average fluorescence intensity value (AFIV), which was calculated from fluorescence intensity values (FIV) of multiple oocytes. The FIV for each oocyte was calculated by subtracting the basal intensity value from the peak value. Each treatment was repeated 3–4 times and each replicate contained about 10 oocytes. (a,b) Values without a common letter above their bars differ significantly (P < 0.05). Photographs D, E and F show ROS levels in GL, GL + D and GL + I oocytes, respectively, when observed under a laser confocal microscope following DCHFDA staining.

The above experiments indicated that blastocyst rates were significantly higher when COCs were matured with nontoxic levels of 1.5 μM iodoacetate than with 150 μM DHEA (Table 2). Since this suggested that PPP played a more important role than glycolysis in promoting ooplasmic maturation, three experiments were performed to explain why PPP is more important than glycolysis. The first experiment tested the effect of inhibiting PPP or glycolysis during oocyte maturation on GSH reserve and redox potential. COCs were matured with 5.6 mM glucose and 0.5 mM lactate with or without 150 μM DHEA or 1.5 μM iodoacetate, and the mature oocytes were assayed for intracellular GSH or ROS. The total GSH (GSX) level decreased significantly in oocytes matured in the presence of either DHEA or iodoacetate compared to that in control oocytes matured without inhibitors (Fig. 1). Whereas the ratio of GSH/GSSG decreased, the level of ROS increased significantly in oocytes matured with DHEA compared to that in control oocytes matured without inhibitors. Neither the GSH/GSSG ratio nor the ROS level differed between oocytes matured with iodoacetate and oocytes in the control group. The results suggested that PPP maintained oocyte redox potential better than glycolysis did.

### Fructose-6-phosphate (F-6-P) can overcome the inhibitory effect of DHEA on ooplasmic maturation

In the second experiment, a hypothesis that PPP might provide intermediate products for glycolysis in cumulus cells was tested. COCs were matured in simplified α-MEM containing 5.6-mM glucose and 0.5-mM lactate with different concentrations of F-6-P with or without 150-μM DHEA or 1.5-μM iodoacetate before observation for maturation and embryo development. Blastocyst rates decreased significantly after maturation with DHEA but without F-6-P (Table 4). After maturation with both DHEA and 10 mM F-6-P, however, blastocyst rates increased to the level in control oocytes matured with neither DHEA nor F-6-P. However, the beneficial effect of F-6-P disappeared completely in the presence of iodoacetate. The results suggested that F-6-P produced by PPP was utilized by glycolysis to support oocyte cytoplasmic maturation.

### Effects of inhibiting PPP or glycolysis during oocyte maturation on intra-oocyte ATP and NADPH contents

**Figure 2:**
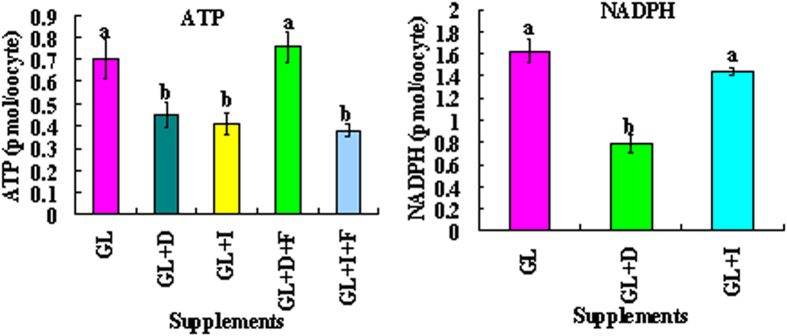
Intra-oocyte ATP and NADPH contents after mouse COCs maturation in simplified α-MEM containing 5.6-mM glucose and 0.5-mM lactate (GL) in the presence (+) or absence of 150-μM DHEA (D) or 1.5-μM iodoacetate (I) and/or 10-mM F-6-P (F). For ATP assays, each treatment was repeated 3 times with each replicate containing 60–80 cumulus-free oocytes, and for NADPH assays, each treatment was repeated 3 times with each replicate containing 100–120 cumulus-free oocytes. (a,b) Values without a common letter above their bars differ significantly (P < 0.05).

In the third experiment, intra-oocyte ATP and NADPH contents were measured following COCs maturation with 5.6-mM glucose and 0.5-mM lactate in the presence or absence of 150-μM DHEA or 1.5-μM iodoacetate and/or 10-mM F-6-P. Treatment with either DHEA or iodoacetate significantly reduced the ATP level (Fig. 2). Supplementation of F-6-P overcame the inhibitory effect of DHEA on ATP production, but had no effect on that of iodoacetate. The results suggested that both PPP and glycolysis produced ATP during oocyte maturation, and that PPP provided F-6-P for glycolysis to generate ATP. Treatment with DHEA significantly reduced the level of NADPH but treatment with iodoacetate showed a mild effect (Fig. 2), suggesting that PPP played a more important role in maintaining oocyte redox potential.

### Mechanism by which pyruvate sustains oocyte cytoplasmic maturation

**Figure 3:**
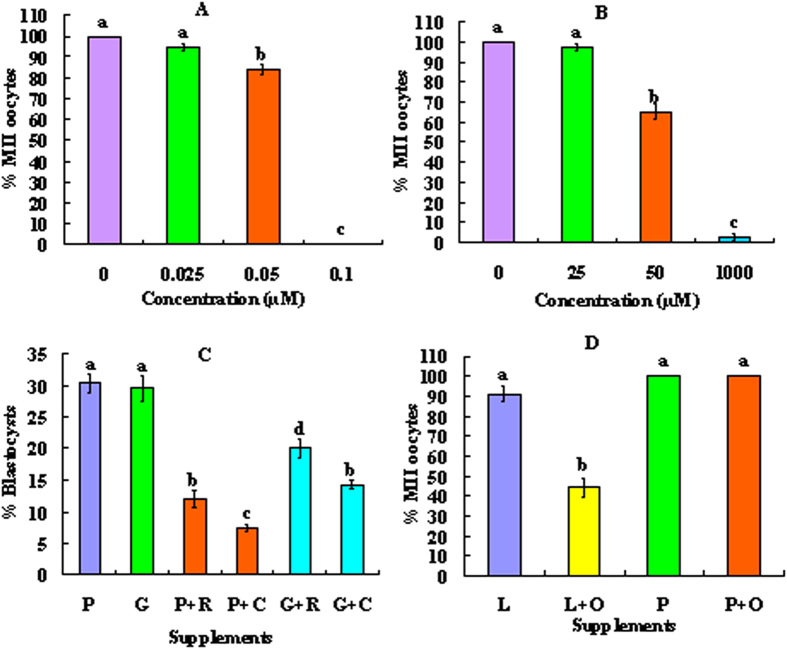
Effects of treatment with rotenone, 4-CIN or oxamate during maturation on maturation and blastocyst formation of mouse oocytes. Panels A and B show percentages of matured (MII) oocytes after COCs were matured with 2-mM pyruvate alone or with different concentrations of rotenone or 4-CIN, respectively. Each treatment was repeated 3 times with each replicate having about 25 oocytes. Panel C shows blastocyst formation after COCs were matured with 2-mM pyruvate (P) or 5.6-mM glucose (G) alone or with (+) 0.025-μM rotenone (R) or 25-μM 4-CIN (C). Each treatment was repeated 4–5 times and each replicate contained around 25 oocytes. Panel D shows percentages of MII oocytes after COCs were matured with 1.5-mM lactate (L) or 2-mM pyruvate (P) alone or in the presence of (+) 30 mM oxamate (O). Each treatment was repeated 3–4 times with each replicate including about 25 oocytes. (a–d) Values without a common letter above their bars differ significantly (P < 0.05).

To study how pyruvate supports ooplasmic maturation, effects of electron transport inhibitor, rotenone, and monocarboxylate transfer inhibitor, 4-CIN, on oocyte maturation were first observed. COCs were matured with 2-mM sodium pyruvate and different concentrations of rotenone or 4-CIN before examination for PB1 extrusion. Percentages of MII oocytes did not differ between maturation without and with 0.025 μM rotenone or 25 μM 4-CIN, but decreased significantly with increasing concentrations (Fig. 3A,B). Thus, 0.025 μM rotenone and 25 μM 4-CIN were selected to observe their effects on ooplasmic maturation. To rule out the possibility that the inhibitors might impair oocyte developmental potential by their toxicity, some COCs were matured with glucose and the inhibitors.

Rates for oocyte maturation, activation and 4-cell embryos did not differ among different treatments (data not shown). Without inhibitors, blastocyst rates did not differ between oocytes matured with pyruvate and oocytes matured with glucose (Fig. 3C), suggesting that glucose metabolism supports ooplasmic maturation mainly by producing pyruvate. Blastocyst rates decreased significantly after COCs maturation with rotenone or 4-CIN in the presence of either pyruvate or glucose. Oocytes matured with rotenone produced significantly more blastocysts than oocytes matured with 4-CIN in the presence of either pyruvate or glucose, suggesting that monocarboxylate transfer plays a more important role than mitochondrial electron transport in ooplasmic maturation. Furthermore, oocytes matured with glucose produced significantly more blastocysts than oocytes matured with pyruvate in the presence of either rotenone or 4-CIN, suggesting that (a) glucose metabolism sustains ooplasmic maturation not only by producing pyruvate but also by other means and (b) the rotenone- or 4-CIN-induced impairment of ooplasmic maturation was not due to drug toxicity.

### Lactate metabolism in cumulus cells supports oocyte maturation via LDH-catalyzed oxidation to pyruvate

To study how lactate in cumulus cells support oocyte maturation, COCs were matured with 2-mM pyruvate or 1.5-mM lactate alone or in the presence of 30 mM oxamate (LDH inhibitor). The results showed that whereas 90% of the oocytes matured after culture with lactate alone, only 44% matured after culture with both lactate and oxamate (Fig. 3D). When COCs were cultured with both pyruvate and oxamate, however, 100% of the oocytes matured, excluding the possibility that oxamate impairs oocyte maturation due to its toxicity. The results suggested that lactate metabolism in cumulus cells supports oocyte maturation via LDH-catalyzed oxidation to pyruvate.

## Discussion

In this study, a maturation medium that could support an acceptable rate of oocyte nuclear maturation but could sustain only a limited blastocyst formation without glucose was first formulated to determine the effect of glucose metabolism on ooplasmic maturation exclusively without involving nuclear maturation. The basic medium we selected for oocyte maturation was a simplified α-MEM that did not contain any energy substance. Because our preliminary results showed that mouse COCs could not mature to the MII stage in the simplified α-MEM alone due to a lack of energy supply, a suitable energy substance at a proper concentration must be used to support oocyte nuclear maturation without improving cytoplasmic maturation. The suitable energy substrate must meet the following two requirements: (a) it did not have any impact on glucose metabolism and (b) it could support a high rate of nuclear maturation but only a limited rate of blastocyst formation when added alone. Among the energy substrates that are most often used in oocyte maturation media, pyruvate was found to interfere with glucose metabolism. For example, addition of glucose to pyruvate-containing medium prevented both the increase in pyruvate consumption and the meiotic induction in mouse COCs^34^,^39^. Similarly, addition of pyruvate to glucose-containing medium resulted in significant reduction in the metabolism of all three glucose analogues^18^.

In contrast, previous studies have suggested that lactate has no effect on glucose metabolism while providing a limited amount of energy in mouse oocytes and early embryos. For instance, a study on the interaction of lactate on pyruvate and glucose metabolism in the early mouse embryos showed that glucose uptake was not affected by lactate in the culture medium^40^. Furthermore, the lactate-derived pyruvate seemed to be minimally metabolized by the mitochondria in mouse oocytes, as mitochondrial oxidation was never affected by lactate addition, even after a delay^41^. According to Dumollard *et al.*^41^, this suggests the presence of discrete pools of pyruvate inside the oocyte: one from the bathing medium, which is rapidly metabolized by the mitochondria, while a second pool derived from lactate is poorly used by the mitochondria. Such intracellular compartmentation of pyruvate pools has also been described in somatic cells^42^,^43^. In this study, when COCs were matured with optimal concentrations of lactate (5 mM) or pyruvate (2 mM), whereas blastocyst rates obtained with pyruvate were as high as those obtained with 5.6 mM glucose, those obtained with lactate were significantly lower. This suggested that whereas the culture medium- and glycolysis-derived pyruvate entered the TCA, the lactate-derived pyruvate was poorly metabolized by the mitochondria of mouse oocytes. Thus, we chose lactate as a supplement to the maturation medium to study the effect of glucose metabolism on ooplasmic maturation. Our results showed that whereas 80% of the COCs completed nuclear maturation, only 10% developed into blastocysts after maturation in the simplified α-MEM containing 0.5 mM lactate. When both 0.5 mM lactate and 5.6 mM glucose were added to the maturation medium, both maturation rate and blastocyst rate were similar to those observed in oocytes matured in the simplified α-MEM supplemented with only 5.6 mM glucose. The results suggested that during maturation of mouse oocytes, lactate did not affect glucose metabolism while providing a certain amount of energy that was enough to support nuclear maturation but was insufficient to sustain full ooplasmic maturation.

The present results showed that the blastocyst rate after oocyte maturation with 1.5 μM iodoacetate (20%) was significantly higher than that after maturation with 150 μM DHEA (12%) (Table 2). This suggested that PPP had played a more important role than glycolysis in promoting oocyte cytoplasmic maturation. Our analysis on the levels of GSH and ROS showed that although the GSX level decreased significantly to the same level whether PPP or glycolysis was inhibited, the level of oxidative stress increased significantly after PPP was inhibited, whereas it remained unchanged following glycolysis inhibition. Once synthesized, GSX cycles between the GSH and the GSSG form through the actions of GSX reductase and GSX peroxidase. The peroxidase transfers electrons from GSH to oxidized molecules within the cytoplasm, minimizing the actions of various oxidative stressors and resulting in the production of GSSG^44^. The cellular pool of GSH is maintained by reduction of GSSG to GSH by GSX reductase as well as further GSH synthesis.

It is known that the synthesis of GSX is dependent on ATP, and the reduction of GSSG requires NADPH^45^,^46^. Our assays of intraoocyte ATP and NADPH indicated that PPP produced similar amount of ATP but significantly more NADPH than glycolysis did during oocyte maturation. Our observations on the effect of F-6-P during oocyte maturation further confirmed that PPP in cumulus cells generated ATP and improved blastocyst development by providing glycolysis with intermediate products like F-6-P. Taken together, the present results suggested that whereas glycolysis promoted ooplasmic maturation mainly by supplying energy, PPP facilitated ooplasmic maturation to a greater extent by both supplying energy and reducing oxidative stress. Our previous study on the effect of glucose metabolism in cumulus cells on oocyte aging have also suggested that glycolysis in cumulus cells might be defective, with pyruvate production depending upon the PPP for intermediate products like F-6-P^44^.

**Figure 4:**
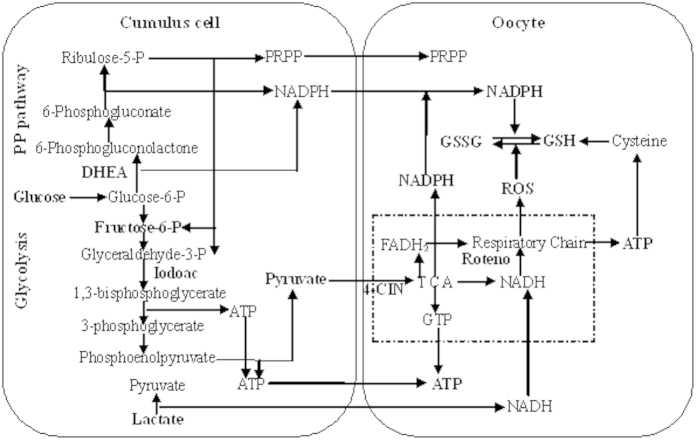
A proposed model for the glucose catabolism in cumulus cells and the maturing oocyte. Whereas the oocyte itself can utilize pyruvate, it cannot use glucose or lactate efficiently to support ooplasmic maturation unless in the presence of cumulus cells. However, the oocyte itself does have the capacity to use lactate for a limited nuclear maturation. Within the cumulus cell, glucose is utilized through PPP and glycolysis. Whereas NADPH and F-6-P/Glyceraldehyde-3-P are produced during PPP, pyruvate and ATP are generated during glycolysis. The PPP-derived F-6-P and Glyceraldehyde-3-P enter glycolysis and produce ATP and pyruvate. Both the glycolysis-derived pyruvate and pyruvate in the medium enters the oocyte and is oxidized by the TCA cycle within the ovum mitochondrion (dotted line box) to generate FADH_2_, NADH, NADPH, and GTP. Lactate in medium enters the cumulus cells and produces NADH via LDH-catalyzed oxidation to pyruvate. Cysteine produced from the reduction of cystine by cysteamine in the oocyte participates in the synthesis of GSH, a process of energy consumption. Whereas NADPH from both PPP and TCA reduces the GSSG, overcoming the oxidizing effect of ROS, NADH produced by TCA and lactate oxidation generates ATP through electron transfer in the respiratory chain. The present results also suggested that whereas the culture medium- and glycolysis-derived pyruvate entered the TCA, the lactate-derived pyruvate was poorly metabolized by the oocyte mitochondria.

The current results showed that oocyte maturation with rotenone produced significantly more blastocysts than maturation with 4-CIN in the presence of either pyruvate or glucose. This suggested that the pyruvate entering the mitochondrion had used means other than the respiratory chain to produce substances beneficial to ooplasmic maturation. It is known that the intra-mitochondrial pyruvate is oxidized by the TCA cycle to generate FADH_2_, NADH, NADPH and GTP (Fig. 4). Among these TCA cycle products, whereas FADH2 and NADH generate ATP through electron transfer in the respiratory chain, GTP produces ATP without involving electron transfer, and NADPH reduces the GSSG, overcoming the oxidizing effect of ROS. In addition, the present results showed that oocyte maturation with glucose produced significantly more blastocysts than maturation with pyruvate in the presence of either rotenone or 4-CIN. It is known that the glucose metabolism pathways upstream pyruvate production also produce beneficial molecules. For example, PPP produces NADPH and PRPP, and glycolysis generates ATP prior to pyruvate generation (Fig. 4). It has been reported that PRPP is involved in purine production and resumption of meiosis^35^,^47^, and that exogenous PRPP promoted oocyte maturation^36^,^48^.

In the current study, the toxicity of DHEA and iodoacetate was tested by the addition of pyruvate, while that for rotenone and 4-CIN was examined by the addition of glucose to the inhibitor-containing maturation medium. The results showed that blastocyst rates did not differ between oocytes matured with pyruvate alone and the oocytes matured with both pyruvate and DHEA or iodoacetate, suggesting that neither inhibitors were toxic to oocytes when used at the levels that had successfully inhibited PPP or glycolysis. Similarly, oocyte maturation with glucose produced significantly more blastocysts than oocyte maturation with pyruvate in the presence of either rotenone or 4-CIN. This suggested that rotenone or 4-CIN used at the selected concentrations were not toxic to oocytes because glucose metabolism upstream the pyruvate production generates molecules other than pyruvate to support ooplasmic maturation as discussed above.

The present results showed that mouse DOs had a limited ability to utilize glucose to support nuclear maturation. Although a few studies reported that mouse DOs metabolized small amount of glucose^18^,^49^,^50^, others demonstrated that they had a limited capacity to use glucose to support nuclear maturation^27^,^28^. It is known that glucose is transported into cells by glucose transporters (GLUTs), which have been found essential for early development^51^,^52^. Han *et al.*^53^ observed that the level of GLUT1 protein was higher significantly in cumulus cells than that in oocytes. In addition, the present study also demonstrated that mouse DOs could not utilize lactate to support nuclear maturation while COCs could use it to support full nuclear maturation and yielded about 20% of blastocysts when added to maturation medium at a higher (1.5 mM) concentration. Eppig^50^ detected little or no evolution of ^14^CO_2_ from mouse DOs of any size incubated in ^14^C-lactate. Cetica *et al.*^54^ found that the addition of lactate to maturation medium failed to improve the maturation rate of bovine DOs. The lactate dehydrogenase (LDH) activity in bovine COCs was much higher than that in the DOs^54^. Our results that only high concentrations of lactate could support a mild ooplasmic maturation of COCs suggested that the lactate-derived pyruvate was poorly metabolized by the mitochondria in cumulus cells and oocyte. We thus proposed that the lactate in cumulus cells promoted ooplasmic maturation mainly by producing NADH through its LDH-catalyzed oxidization to pyruvate. The NADH then entered the oocyte and generated ATP through electron transfer in the respiratory chain (Fig. 4). Our experiment of LDH inhibition with oximate further confirmed that lactate metabolism in cumulus cells supports oocyte maturation via LDH-catalyzed oxidation to pyruvate.

In summary, a maturation medium that could support oocyte nuclear maturation in the absence of glucose but sustained only a limited blastocyst formation without glucose was first formulated in this study. By manipulating glucose metabolism pathways using such a maturation medium, we have studied the effect of the glucose metabolism pathways on ooplasmic maturation. The results suggest that glucose metabolism plays a crucial role in ooplasmic maturation. The culture system we established can be successfully used for study of glucose metabolism of oocytes without using drugs to control meiotic resumption. The data obtained not only have contributed to our understanding of the glucose metabolism in oocytes but also have provided important information that can potentially be used for the formulation of more optimal media in clinical assisted reproductive technology.

## Methods

The experimental procedures used for animal care and handling were approved by the Animal Care and Use Committee of the Shandong Agricultural University P. R. China (Permit number: SDAUA-2001-001). The methods were carried out in accordance with the approved guidelines. Unless otherwise specified, all chemicals and reagents used were purchased from Sigma Chemical Co. (St. Louis, MO, USA).

### Animals and oocyte recovery

Mice of the Kun-Ming breed were kept in a room with 14 L: 10 D cycles, the dark starting from 20:00 h. Female mice 8–10 weeks after birth were killed at 48 h after intra-peritoneal injection of 10 IU equine chorionic gonadotropin (eCG) per mouse (Ningbo Hormone Product Co. Ltd., Cixi City, P.R. China), and the large follicles on the ovary were ruptured in M2 medium to release cumulus-oocyte-complexes (COCs) at the GV stage. Only COCs with more than three layers of unexpanded cumulus cells and containing oocytes >70 μm in diameter with a homogenous cytoplasm were selected. To prepare cumulus-denuded oocytes (DOs), cumulus cells were removed from some of the selected COCs mechanically by pipetting with a small-bore pipette.

### *In vitro* maturation of oocytes

The maturation medium used was α-MEM simplified by removing all vitamins, amino acids (except glutamine) and nucleosides. The simplified α-MEM thus contained inorganic salts (1.8 mM CaCl2, 0.81 mM MgSO4, 5.3 mM KCl, 26.2 mM NaHCO_3_, 117.2 mM NaCl, 1.0 mM NaH_2_PO_4_), 2 mM glutamine, 4 mg/ml bovine serum albumin, 10 IU/ml eCG, 0.03 mM phenol red, 50 IU/ml penicillin, and 50 μg/ml streptomycin. Depending on the experiment, different concentrations of energy substrates and metabolism regu0lators were added to the maturation medium. To prepare stock solutions, dehydroepiandrosterone (DHEA, 200 mM), a-cyano-4-hydroxy cinnamate (4-CIN; 100 mM) and rotenone (1 mM) were dissolved in dimethyl sulfoxide; iodoacetate (4 mM) was dissolved in water. All the stock solutions were stored in aliquots at −20 °C and diluted to desired concentrations with the maturation medium immediately before use. Sodium oxamate and disodium fructose-6-phosphate (F-6-P) was dissolved directly in maturation medium before use. The osmotic pressure of the medium was adjusted by decreasing the amount of sodium chloride accordingly when sodium lactate, sodium pyruvate, disodium F-6-P and/or sodium oxamate was included in the medium. After being washed three times in M2 and once in the maturation medium, the recovered oocytes were cultured for 15 h in groups of around 25 in 100 μl of maturation medium at 37.5 °C under 5% CO_2_ in humidified air. At the end of *in vitro* maturation, oocytes were allocated to *in vitro* aging or assays for intra-oocyte concentrations of glutathione (GSH), reactive oxygen species (ROS), ATP or NADPH.

### *In vitro* aging of oocytes

The medium used for oocyte aging was the Chatot-Ziomek-Bavister (CZB) medium (NaCl, 81.62 mM; KCl, 4.83 mM; KH_2_PO_4_, 1.18 mM; MgSO_4_, 1.18 mM; NaHCO_3_, 25.12 mM; CaCl_2_, 1.7 mM; ethylenediaminetetra-acetic acid [EDTA], 0.11 mM; glutamine, 1 mM; bovine serum albumin, 5 g/L; penicillin, 0.06 g/L; streptomycin, 0.05 g/L). After being washed three times in M2 medium and once in the aging medium, oocytes were cultured for 6 h in 100 μl of aging medium at 37.5 °C under 5% CO_2_ in humidified air. At the end of *in vitro* aging, the oocytes were subjected to parthenogenetic activation.

### Oocyte activation and embryo culture

At 6 h of aging, oocytes were stripped of their cumulus cells (if any) by pipetting in M2 containing 0.1% hyaluronidase. After being washed twice in M2 and once in activating medium (Ca^2+^-free CZB medium supplemented with 10 mM SrCl_2_ and 5 μg/ml cytochalasin B), the oocytes were incubated in the activating medium for 6 h at 37.5 °C in a humidified atmosphere with 5% CO_2_ in air. At the end of the activation treatment, the oocytes were examined with a microscope for the evidence of activation. Oocytes were considered activated when they contained one or two well-developed pronuclei. Activated oocytes were cultured for 4 days in regular CZB at 37.5 °C under humidified atmosphere with 5% CO_2_ in air. Glucose (5.5 mM) was added to CZB when the embryos were cultured beyond the 3- or 4-cell stage.

### Assay for intracellular GSH

Cumulus-free matured oocytes were washed three times in M2 medium. Five microliters of distilled water containing 20–30 oocytes was transferred to a 1.5-ml microfuge tube, and then 5 μl of 1.25 M phosphoric acid were added to the tube. Samples were frozen at −80 °C and thawed at room temperature. This procedure was repeated three times. Next, the samples were stored at −20 ° until analyzed. Concentrations of total GSH (GSX) in the oocyte were determined by the 5,5’-dithio-bis (2-nitrobenzoic acid) (DTNB)-oxidized GSH (GSSG) reductase-recycling assay. Briefly, 700 μl of 0.33 mg/ml of NADPH in 0.2 M sodium phosphate buffer containing 10 mM EDTA (stock buffer, pH 7.2), 100 μl of 6 mM DTNB in the stock buffer, and 190 μl of distilled water were added and mixed in a microfuge tube. Ten microliters of GSH reductase (G-3664; 250 IU/ml) were added with mixing to initiate the reaction. The absorbance was monitored continuously at 412 nm with a spectrophotometer for 3 min, with a reading recorded every 0.5 min. To measure the concentrations of GSSG, the samples (10 μl) were vigorously mixed with 0.2 μl of 2-vinylpyridine and 0.6 μl of triethanolamine. After 60 min, the sample was assayed as described above in the DTNB-GSSG reductase-recycling assay. Standards (0.01, 0.02, 0.1, 0.2, and 1.0 mM) of GSX and a sample blank lacking GSX were also assayed. The amount of GSX in each sample was divided by the number of oocytes to get the intracellular GSX concentration per oocyte (pmol/oocyte). The reduced GSH (GSH) values were calculated from the difference between GSX and GSSG for each oocyte.

### Assay for intraoocyte ROS

In order to quantify ROS in individual oocytes, intraoocyte H_2_O_2_ levels were measured using 2′,7′-dichlorodihydrofluorescein diacetate (DCHFDA). Stock solution of DCHFDA was prepared in dimethyl sulfoxide at 1 mM and stored in the dark at −20 °C. Immediately before use, the stock solution was diluted to 0.01 mM in M2. Cumulus-free oocytes were stained for 10 min with the DCHFDA solution. After being washed thoroughly to remove the traces of the dye, about 10 oocytes were placed on a slide, covered with a coverslip, and observed under a Leica laser scanning confocal microscope. The fluorescence was obtained by excitation at 488 nm. Photographs were taken using fixed microscopic parameters, and the fluorescence intensity from each oocyte was analyzed using a Leica software.

### Assay for intraoocyte ATP

To determine ATP within oocytes, 300 μl of hot distilled water containing 60–80 cumulus-free oocytes was transferred to a glass homogenizer and homogenized manually. The homogenate was incubated in boiling water for 10 min, vortexed for 1 min, centrifuged for 5 min, and finally, the supernatant was collected. The concentrations of ATP were determined using colorimetric methods with a spectrophotometer (Beckman Coulter DU 800). The ATP assay kit (A095) used was purchased from Nanjing Jiancheng Institute of Bioengineering (Nanjing, Jiangsu Province, China) and the assays were conducted following the instructions of the kits. The amount of ATP in each sample was divided by the number of oocytes to get the intracellular ATP concentration per oocyte (pmol/oocyte).

### Assay for intraoocyte NADPH

Intraoocyte NADPH content was measured using a commercial NADPH assay kit (A115, Nanjing Jiancheng Bioengineering Institute, Jiangsu Province, China) and a spectrophotometer (Beckman Coulter DU 800). One hundred microliters of alkaline extract containing 100–120 cumulus-free oocytes was transferred to a 1.5-ml microfuge tube. After ultrasonication, boiling, ice bath cooling and centrifugation, the supernatant was mixed and neutralized with the same volume of acid extract. After centrifugation, the supernatant was placed on ice until analysis. Colorimetric NADPH assays were performed following the manufacturer’s protocol. The amount of NADPH in each sample was divided by the number of oocytes to get the intracellular NADPH concentration per oocyte (pmol/oocyte).

### Statistical analysis

There were at least three replicates for each treatment unless otherwise stated. Percentage data were arc sine-transformed and analyzed with ANOVA. A Duncan multiple comparison test was used to find differences during ANOVA. The software used was Statistics Package for Social Sciences (SPSS 11.5, SPSS, Inc.). Data are expressed as means ± S.E.M., and P < 0.05 was considered significant.

## Additional Information

**How to cite this article**: Xie, H.-L. *et al.* Effects of glucose metabolism during *in vitro* maturation on cytoplasmic maturation of mouse oocytes. *Sci. Rep.* **6**, 20764; doi: 10.1038/srep20764 (2016).

## Figures and Tables

**Table 1:** Oocyte maturation and embryo development after mouse COCs or DOs were matured in simplified α-MEM supplemented with different concentrations of glucose (G), pyruvate (P) or lactate (L), or both lactate and glucose (L+G).

G/L/P	mM	Oocyte type	% (No.) GVBD Oocytes^1^	% (No.) MII oocytes^2^	% (No.) Blastocysts /activated oocytes^3^
—	0	COCs	48.0 ± 10.1 (36/75)^b^	17.7 ± 3.8 (13/74)^c^	—
—	0	DOs	7.1 ± 2.5 (5/63)^d^	0.0 ± 0.0 (0/11)^d^	—
G	0.56	COCs	—	81.3 ± 1.3 (61/75)^b^	—
**G**	**5.6**	**COCs**	**100.0 ± 0.0 (78/78)**^**a**^	**97.6 ± 1.6 (120/123)**^**a**^	**34.2 ± 0.9 (38/111)**^**a**^
G	5.6	DOs	6.4 ± 3.2 (4/68)^d^	0.0 ± 0.0 (0/19)^d^	—
L	0.25	COCs	—	41.9 ± 1.2 (31/74)^c^	—
**L**	**0.5**	**COCs**	**90.7 ± 5.8 (75/75)**^**a**^	**78.4 ± 4.5 (90/115)**^**b**^	**10.1 ± 1.2 (8/78)**^**b**^
L	1.5	COCs	—	93.3 ± 2.7 (70/75)^a^	23.0 ± 2.6 (22/96)^c^
L	5	COCs	—	100.0 ± 0.0 (127/127)^a^	21.2 ± 1.7 (23/110)^c^
L	0.5	DOs	25.5 ± 4.4 (19/75)^c^	0.0 ± 0.0 (0/64)^d^	—
L	3	DOs	—	1.2 ± 1.2 (1/74)^d^	—
L	30	DOs	—	4.0 ± 4.0 (3/71)^d^	—
**G + L**	**5.6 + 0.5**	**COCs**	**100.0 ± 0.0 (80/80)**^**a**^	**98.7 ± 1.3 (146/148)**^**a**^	**31.9 ± 2.1 (39/123)**^**a**^
G + L	5.6 + 0.5	DOs	26.3 ± 3.5 (19/71)^c^	0.0 ± 0.0 (0/56)^d^	—
P	1	COCs	—	98.3 ± 1.7 (122/124)^a^	17.9 ± 1.3 (18/102)^bc^
P	2	COCs	100.0 ± 0.0 (75/75)^a^	100.0 ± 0.0 (148/148)^a^	29.9 ± 1.7 (38/128)^a^
P	2	DOs	100.0 ± 0.0 (135/135)^a^	100.0 ± 0.0 (135/135)^a^	8.5 ± 1.0 (10/118)^b^

a–d: Values without a common letter in their superscripts differ (P < 0.05) in the same column.

^1^GVBD was examined at 5 h of maturation culture.

^2^Because there are often significant differences between treatments in the viability of the oocytes when energy substrates are manipulated, percentages of GVBD and MII oocytes were calculated only from survived oocytes.

^3^Percentages of activated oocytes and 4-cell embryos did not differ among different treatments (data not shown).

**Table 2:** Oocyte maturation and embryo development after COCs were matured in simplified α-MEM supplemented with 5.6 mM glucose, 0.5 mM lactate and different concentrations of DHEA or iodoacetate.

DHEA / Iodoacetate (μM)	% (No.) MII oocytes	% (No.) Activated oocytes	% (No.) 4-cell embryos	% (No.) Blastocysts
DHEA				
0	100.0 ± 0.0 (124/124)^a^	92.6 ± 4.6 (115/124)^a^	83.9 ± 4.4 (96/115)^a^	29.4 ± 1.2 (34/115)^a^
100	97.5 ± 1.0 (118/121)^a^	89.0 ± 3.6 (105/118)^a^	84.1 ± 3.7 (89/105)^a^	23.0 ± 1.2 (24/105)^b^
150	82.5 ± 5.3 (96/117)^b^	87.6 ± 5.3 (85/96)^a^	75.4 ± 2.9 (64/85)^a^	11.6 ± 1.1 (10/85)^c^
200	80.6 ± 2.7 (130/162)^b^	92.6 ± 4.1 (117/130)^a^	74.1 ± 3.3 (88/117)^a^	10.0 ± 1.4 (12/117)^c^
Iodoacetate				
0	100.0 ± 0.0 (124/124)^a^	92.8 ± 3.9 (115/124)^a^	83.5 ± 6.8 (97/115)^a^	33.2 ± 1.6 (38/115)^a^
1	100.0 ± 0.0 (148/148)^a^	96.6 ± 1.6(143/148)^a^	83.9 ± 4.1(120/143)^a^	32.1 ± 1.6 (46/143)^a^
1.5	100.0 ± 0.0 (121/121)^a^	91.6 ± 3.5 (111/121)^a^	84.7 ± 2.6 (94/111)^a^	20.0 ± 1.4 (22/111)^b^
2	95.5 ± 2.3 (132/139)^a^	94.8 ± 3.4 (126/132)^a^	57.2 ± 5.2 (71/126)^b^	9.6 ± 1.4 (12/126)^c^

a–c: Values without a common letter in their superscripts differ (P < 0.05) in the same column.

**Table 3:** Oocyte maturation and embryo development after COCs were matured in simplified α-MEM containing 2-mM pyruvate (P) alone or with different concentrations of DHEA (D) or iodoacetate (I).

Supplements	% (No.) MII oocytes	% (No.) Activated oocytes	% (No.) 4-cell embryos	% (No.) Blastocysts
P	97.6 ± 2.4 (121/124)^a^	82.3 ± 4.8 (100/121)^a^	76.0 ± 4.8 (76/100)^a^	29.9 ± 2.6 (30/100)^a^
P + D (150μM)	100.0 ± 0.0 (120/120)^a^	92.7 ± 5.3 (111/120)^a^	82.7 ± 3.7 (95/111)^a^	32.3 ± 2.5 (36/111)^a^
P + I (1.5μM)	100.0 ± 0.0 (118/118)^a^	88.4 ± 3.7 (106/118)^a^	87.1 ± 2.5 (93/106)^a^	30.6 ± 2.0 (33/106)^a^
P + I (2 μM)	100 ± 0.0 (125/125)^a^	85.6 ± 3.7 (107/125)^a^	77.8 ± 2.4 (83/107^)a^	20.5 ± 2.0 (22/107)^b^
P + I (3 μM)	96.8 ± 2.3 (113/117)^a^	93.8 ± 1.8 (106/113)^a^	59.5 ± 6.5 (63/106)^b^	2.8 ± 1.1 (3/106)^c^

a–c: Values without a common letter in their superscripts differ (P < 0.05) in the same column. Pyruvate was used at the optimal concentration (2-mM) selected in a preliminary experiment (data not shown).

**Table 4:** Oocyte maturation and embryo development after COCs were matured in simplified α-MEM containing 5.6-mM glucose and 0.5-mM lactate in the absence or presence of 150-μM DHEA or 1.5-μM iodoacetate and different concentrations of F-6-P.

DHEA	F-6-P (mM)	Iodoacetate	% (No.) MII oocytes	% (No.) 4-cell embryos	% (No.) Blastocysts
—	0	—	98.6 ± 0.9 (141/143)^a^	80.7 ± 4.4 (102/126)^a^	30.4 ± 2.6 (38/126)^a^
+	0	—	82.9 ± 1.8 (127/153)^c^	77.5 ± 3.5 (86/112)^a^	12.1 ± 1.5 (13/112)^b^
+	10	—	91.8 ± 3.2 (135/147)^a,b^	80.7 ± 3.5 (101/126)^a^	29.4 ± 1.8 (37/126)^a^
+	20	—	90.8 ± 2.7 (134/147)^b^	64.6 ± 5.5 (76/117)^b^	6.0 ± 0.7 (7/117)^c^
+	10	+	81.0 ± 2.7 (128/158)^c^	75.0 ± 2.8 (88/116)^a,b^	10.3 ± 1.1 (12/116)^b,c^

a–c: Values without a common letter in their superscripts differ (P < 0.05) in the same column. Percentages of activated oocytes did not differ among different treatments (data not shown).
